# The Coat Protein of Citrus Yellow Vein Clearing Virus Interacts with Viral Movement Proteins and Serves as an RNA Silencing Suppressor

**DOI:** 10.3390/v11040329

**Published:** 2019-04-05

**Authors:** Atta Ur Rehman, Zhuoran Li, Zuokun Yang, Muhammad Waqas, Guoping Wang, Wenxing Xu, Feng Li, Ni Hong

**Affiliations:** 1 Key Lab of Plant Pathology of Hubei Province, College of Plant Science and Technology, Huazhong Agricultural University, Wuhan 430070, China; attaehsan4u@yahoo.com (A.U.R.); hzauyangzk@163.com (Z.Y.); muhammadwaqas223@gmail.com (M.W.); gpwang@mail.hzau.edu.cn (G.W.); xuwenxing@mail.hzau.edu.cn (W.X.); 2 Key Laboratory of Horticultural Crop (Fruit Trees) Biology and Germplasm Creation of the Ministry of Agriculture, Wuhan 430070, China; 3 Plant Pathology Section, Central Cotton Research Institute, Sakrand, Sindh 67210, Pakistan; 4 Key Laboratory of Horticultural Plant Biology (MOE), College of Horticulture and Forestry Sciences, Huazhong Agricultural University, Wuhan 430070, China; 18986173968@163.com

**Keywords:** citrus yellow vein clearing virus, coat protein, triple gene block proteins, protein-protein interaction, subcellular localization, RNA silencing suppressor

## Abstract

*Citrus yellow vein clearing virus* is a newly accepted member of the genus *Mandarivirus* in the family *Alphaflexiviridae*. The triple gene block proteins (TGBp1, TGBp2 and TGBp3) encoded by plant viruses in this family function on facilitating virus movement. However, the protein function of citrus yellow vein clearing virus (CYVCV) have never been explored. Here, we showed in both yeast two-hybrid (Y2H) and bimolecular fluorescence (BiFC) assays that the coat protein (CP), TGBp1 and TGBp2 of CYVCV are self-interacting. Its CP also interacts with all three TGB proteins, and TGBp1 and TGBp2 interact with each other but not with TGBp3. Furthermore, the viral CP colocalizes with TGBp1 and TGBp3 at the plasmodesmata (PD) of epidermal cells of *Nicotiana benthamiana* leaves, and TGBp1 can translocate TGBp2 from granular-like structures embedded within ER networks to the PD. The results suggest that these proteins could coexist at the PD of epidermal cells of *N. benthamiana*. Using *Agrobacterium* infiltration-mediated RNA silencing assays, we show that CYVCV CP is a strong RNA silencing suppressor (RSS) triggered by positive-sense green fluorescent protein (GFP) RNA. The presented results provide insights for further revealing the mechanism of the viral movement and suppression of RNA silencing.

## 1. Introduction

A large group of plant viruses possess a triple gene block (TGB) encoding three proteins (TGBp1, TGBp2 and TGBp3) that coordinately function in facilitating cell-to-cell and long-distance movement of plant viruses [1,2,3]. Viruses possessing TGBps have been categorized into two major classes, i.e., hordei-like and potex-like viruses, according to their differences in the mechanism involved in virus movement [4,5,6,7]. Potex-like viruses include viruses in the genera *Potexvirus*, *Allexivirus*, and *Mandarivirus* of the family *Alphaflexiviridae* and the genera *Carlavirus* and *Foveavirus* of the family *Betaflexiviridae.* Virions of potex-like viruses are filamentous, contain a monopartite RNA genome and need coat protein (CP) for their movement from one cell to another. Hordei-like viruses consist of species in the genera *Hordeivirus*, *Pomovirus*, *Pecluvirus*, and *Benyvirus* [7] and form rod-shaped virions containing multipartite genomic RNA segments; their CPs are not necessarily involved in viral cell-to-cell movement [8]. Additionally, potex-like TGBp1 proteins differ substantially from hordei-like TGBp1 proteins by having a lower molecular mass (~25 kDa vs. 42–63 kDa) and lacking an N-terminal domain (NTD) and an internal domain (ID) [4,9]. However, both potex-like and hordei-like TGBp1 proteins contain a helicase-like domain (HELD) consisting of 25 amino acids at the N-terminus that includes three conserved arginine residues necessary for its functions. Until now, most studies on the functions of TGB proteins have been performed for two potexviruses, potato virus X (PVX), and bamboo mosaic virus (BaMV) [10]. The TGBp1 of PVX and BaMV localizes to the cytoplasm and nuclei as inclusions in BaMV- and PVX-infected plant tissues, respectively [11,12]. Their TGBp1 has helicase activity [13] and the ability to increase the size exclusion limit (SEL) of plasmodesmata (PD) [14,15,16,17,18] and to promote the translation of virus-derived RNAs [19,20]. TGBp2 is membrane-associated and can form endoplasmic reticulum (ER) granular vesicles [12,21,22], TGBp3 contains a single N-terminal transmembrane (TM) domain and might be ER-associated [23]. The interactions of TGB proteins and CP of potexviruses play important roles in virus movement and have been investigated by several research groups [3,5,10,12,14,24]. The PD and/or ER locations of these proteins and the formation of the TGB proteins-virion complex are necessary for intracellular viral transport [25,26,27]. The cooperation between two TGB proteins and the CP of cymbidium mosaic potexvirus (CymMV) is a crucial determinant for viral systemic movement in *Nicotiana benthamiana* [28].

In addition to serving as a structural component to protect viral genomic RNA(s) and being involved in virus movement, the CPs of many plant viruses have multiple functions during plant-virus interactions as outlined recently [29]. The CP of pepino mosaic potexvirus (PepMV) serves as an efficient RNA silencing suppressor (RSS) with diverse functions [24]. CP (p38) of turnip crinkle virus (TCV) may suppress RNA silencing at multiple levels, such as preventing vsiRNA generation and blocking activity of assembled RNA-induced silencing complex (RISC) [30]. The CP (p37) of pelargonium line pattern virus (PLPV) functions as a RSS through siRNA sequestration [31]. For potexviruses, the TGBp1 of BaMV and PVX is implicated in suppressing virus-induced gene silencing (VIGS) in host cells [32,33,34]. *Citrus yellow vein clearing virus* is a recently described member of the genus *Mandarivirus* in the family *Alphaflexiviridae* [35]. The citrus yellow vein clearing virus (CYVCV) is a supposedly new threat to the citrus fruit industry [36]. CYVCV-infected citrus trees decline quickly and show vein yellowing and clearing, leaf distortion, and occasional ringspots and venial necrosis [37,38]. CYVCV possesses a 7.5 kb single-stranded positive-sense RNA genome consisting of six open reading frames (ORFs) and is 5’-capped and 3’-polyadenylated. ORF1 (4.7 kb) encodes a 187.3 kDa replicase polyprotein; ORFs 2-4 encode TGBps with molecular weights of 25 kDa (TGBp1), 12 kDa (TGBp2) and 6.4 kDa (TGBp3); ORF5 encodes a 35 kDa coat protein (CP) and ORF6 encodes a 23 kDa protein (23K) with unknown function [37,39]. Viruses in the genus *Mandarivirus* possess TGB proteins with sizes and conserved domains similar to those of potexviruses. However, the interactions among TGB proteins and CPs of mandariviruses, their involvement in viral movement and their possible role in posttranscriptional gene silencing suppression have never been studied. In CYVCV-infected plants, the accumulation of siRNA has been reported [40], but a CYVCV-encoded suppressor has never been identified. This study aims to investigate interactions of CYVCV proteins, their subcellular localization and potential viral RSS. Here, for the first time, we report that the CP of CYVCV, a mandarivirus protein, functions as a local RSS.

## 2. Materials and Methods

### 2.1. Virus Source and Plant Material

Eureka lemon (*Citrus limon* (L.) Burm. f.) plants infected by CYVCV (isolate HB) were kept in an insect-proof greenhouse and used as the virus source. Plants of *N. benthamiana* (wild-type and 16c) were grown in a growth chamber in 60% relative humidity with a 16 h photoperiod at 25 °C during the day and an 8 h dark period at 20 °C during the night.

### 2.2. Gene Cloning

Total RNA was extracted from leaves of a CYVCV-infected Eureka lemon plant by using TRIzol reagent (Invitrogen, Carlsbad, CA, USA). Reverse transcription was performed by using Maloney murine leukemia virus (M-MLV) reverse transcriptase (Promega, Madison, WI, USA) and a random primer hexadeoxyribonucleotide mixture pd(N)6 (TaKaRa, Dalian, China) at 37 °C for 1.5 h. Primers used for the amplification of five ORFs encoding proteins TGBp1, TGBp2, TGBp3, CP, and 23K of CYVCV were designed based on multiple alignments of the viral sequences available in GenBank (Table S1). The PCR products were gel purified and ligated into a pMD18-T vector (TaKaRa, Dalian, China). For each amplified fragment, at least three clones were sequenced at Shanghai Sangon Biological Engineering and Technology and Service Co. Ltd., China. One clone of each amplicon with a consensus sequence was selected for further study. The nucleotide and amino acid sequence alignments were carried out by using the MView online tool with default settings. The corresponding sequences of CYVCV isolates CYVCV-Y1 (JX040635), CYVCV-RL (KP120977) and CYVCV-PK (KP313241) available in GenBank were included in the analysis.

### 2.3. Yeast Two-Hybrid Assays

Yeast two-hybrid (Y2H) assays were carried out using the Matchmaker^TM^ Gold Yeast Two-Hybrid System (Clontech, Mountain View, CA, USA) according to the manufacturer’s protocol. The cloned full-length ORFs were excised from the recombinant pMD18-T plasmid using restriction enzymes (double enzymatic digestion), gel purified and ligated into the yeast shuttle vectors pGBKT7 (BD) and pGADT7 (AD) (Clontech, Mountain View, CA, USA) digested with corresponding enzymes. Constructs were transformed into *Saccharomyces cerevisiae* (strain Y2HGold) according to the Matchmaker System 2 protocol (Clontech, Mountain View, CA, USA). Yeast cell transformations for each pair of plasmids were performed with an equal amount of bait and prey plasmids. Transformed yeast cells were streaked on double dropout agar (SD/-Leu/-Trp) plates with high rigor supplemented with X-α-Gal (20 mg/mL) and Aureobasidin A (500 µg/mL), and the plates were incubated for three days at 30 °C. Colonies that turned to blue were further plated on quadruple dropout agar (SD/-Leu/-Trp/-His/-Ade/X-α-Gal/AbA). The positive and negative interactions were evaluated based on blue colonies on the QDO/X/AbA medium. *S. cerevisiae* cells were cotransformed with pGBKT7-Lam and pGADT7-T, and pGBKT7-p53 and pGADT7-T were used as a negative and a positive control, respectively. Autoactivation tests were conducted for all constructs as described in the Matchmaker System 2 protocol (Clontech, Mountain View, CA, USA).

### 2.4. BiFC Assay and Subcellular Localization

For the BiFC assays, the targeted genes were individually inserted into plant expression vectors using GatewayTM technology (Invitrogen, Carlsbad, CA, USA). To facilitate subsequent gateway cloning, primer sequences were flanked with attB recombination sites at their 5′ ends (Table S2). The PCR products containing entire attB sites were purified and recombined into the pDONR/Zeo vector with the Gateway™ BP Clonase™ Enzyme Mix according to the manufacturer’s recommendations. Sequences of the entry clones were verified before subsequent steps. The entry clones containing each of the candidate genes were recloned into the pEarlygate202-YN and pEarlygate201-YC vectors using the Gateway™ LR Clonase™ II Enzyme mix. The YFP-fusion constructs were transformed into *Agrobacterium tumefaciens* strain GV3101, then agroinfiltrated pairwise into the epidermal cells of *N. benthamiana* leaves.

For subcellular localization experiments, (35S)2-MCS-eYFP (pCNY)- and (35S)2-MCS-eCFP (pCNC)-expressing constructs were generated. Full-length eYFP and eCFP were amplified and ligated into the *Kpn*I-*Sma*I site of pCNF3 to generate pCNY and pCNC constructs, respectively. Target genes were amplified from the corresponding clones by using primers with an *Xba*I or *Bam*HI digestion site (Table S3). The *Xba*I- and *Bam*HI-digested TGBp1, TGBp2, TGBp3, and CP coding sequences were introduced into pCNY and pCNC, respectively. Binary constructs for expression of mCherry-HDEL fused to mRFP was used as an ER marker [41], and H2B and CMV 3a were used as a nuclear and a PD marker, respectively. These constructs were transformed into *A. tumefaciens* (strain GV3101) by the heat shock method. The resulting fusion proteins were transiently expressed in leaves of *N. benthamiana* plants using an agroinfiltration method to determine intracellular localization and interactions. *Agrobacterium* cultures were centrifuged at 3500 rpm, and pellets were resuspended in 10 mM 2-(4-morpholino) ethanesulfonic acid (MES) solution (pH 5.85) containing 10 mM MgCl_2_ and 150 mM acetosyringone and then incubated at room temperature for 2–3 h. *Agrobacterium* suspensions at 0.5–1.0 optical density (OD600) harboring the constructs were infiltrated or coinfiltrated into *N. benthamiana* leaves (five weeks old) using a 1 mL needleless syringe.

The above leaf sections infiltrated with *Agrobacterium tumefaciens* cultures were visualized for florescence signals using confocal laser scanning microscopy (CLSM; Leica Microsystems, TCS-SP8, Germany) with an HC PL APO CS2 63x/1.20 WATER objective at 2–4 days post infiltration (dpi).

### 2.5. Identification of Viral RSS

To determine local RNA silencing suppression activity, the *Xba*I- and *Bam*HI-digested TGBp1, TGBp2, TGBp3, CP and 23K genes were inserted into the *Xba*I and *Bam*HI sites of pCNF3-Flag-tagged with a CaMV 35S promoter. For detecting systemic suppression of RNA silencing, CYVCV genes were amplified using primers listed in Table S4 and inserted in the vector spdk-ΔTGBΔCP-Flag. The vectors were constructed by using the ClonExpress II One Step Cloning Kit (Vazyme Biotech Co., Ltd.). Each recombinant vector was transformed into *Agrobacterium tumefaciens* (strain GV3101) by heat shock. An *Agrobacterium* culture containing plasmid pMS4 (with a 35S-gfp) was used as an inducer of RNA silencing. The well-known suppressor P19 of tomato bushy stunt virus (TBSV) was used as a positive control. *Agrobacterium* cultures individually transformed with each of the constructs and cultures carrying pMS4 were coinfiltrated into the leaves of wild-type *N. benthamiana* for local silencing tests and of GFP-expressing *N. benthamiana* 16c for systemic silencing tests at a ratio of 1:1 (*v/v*). Each assay was repeated at least three times. Local GFP expression signals were monitored at 3–6 dpi, and systemic GFP expression signals were monitored at 20 dpi under a hand-held UV lamp (LUYOR^®^-3104). Images were captured using Canon EOS 450D camera (Canon Inc., Tokyo, Japan). 

### 2.6. Western Blot Analysis

Western blotting was performed to validate the expression levels of target proteins. Genes encoding the five proteins mentioned above were fused into the flag-tagged expression vector pCNF3. Total proteins extracted from infiltrated leaf patches of *N. benthamiana* were separated by 10% sodium dodecyl sulfate-polyacrylamide gel electrophoresis (SDS-PAGE) and transferred onto a polyvinylidene difluoride (PVDF) membrane (BIORAD, Hercules, USA). The membrane was blocked in 5% nonfat dry milk in Tris-buffered saline (TBS). The monoclonal antibody tag anti-DYKDDDDK (Transgene-Biotech, Beijing, China) at a dilution of 1:5000 was used to detect Flag-tagged proteins, and then the membrane was incubated with peroxidase-conjugated goat-anti-mouse IgG (H + L) (Transgene-Biotech, Beijing, China) at a dilution of 1:5000. Blot signals were developed using a Phototope-Star detection kit and captured by using a Molecular Imager^®^ ChemiDoc^TM^ XRS. Densitometry.

### 2.7. Small Interference RNA Hybridization

Total RNA was extracted from a pool of 5–8 agroinfiltrated leaves using TRI (Sigma-Aldrich, St. Louis MO, USA) according to the manufacturer’s instructions (Invitrogen, Carlsbad, CA, USA). RNAs were separated on vertical gels consisting of 12% polyacrylamide and 8 M urea in the presence of 1× TBE. RNA was transferred onto a Hybond-N membrane (GE Healthcare, Little Chalfont, Bucks, UK) in the presence of 20× SSC by capillary blotting overnight. The RNA was cross-linked to the membrane in a UV Stratalinker 1800 (Stratagene, Maryland, USA). The membrane was prehybridized in PerfectHyb (Sigma-Aldrich, St. Louis MO, USA) for 1 h at 37 °C and then hybridized with [a-^32^P]UTP-labeled antisense GFP RNA at 37 °C overnight [42]. The membrane was washed three times in 1× SSC and 0.1% SDS at 37 °C. An Amersham Typhoon biomolecular imager (Amersham Pharmacia, Piscataway, NJ, USA) was used to detect the hybridization signals.

### 2.8. In Silico Analysis of Protein Sequences

Predictions for nuclear localization and export signals of CYVCV proteins were performed by using the cNLS Mapper online tool [43]. Secondary and tertiary structures were generated with the Phyre server (http://www.sbg.bio.ic.ac.uk/phyre2) [44]. Three-dimensional structures were modeled with Modeller v9.11. The ClusPro2 server was used to perform the interaction analysis. Transmembrane helices/domains were predicted through the ΔG prediction online server (http://dgpred.cbr.su.se/) [45] and TOPCONS (http://topcons.net/) [46].

## 3. Results

### 3.1. Characterization of CYVCV Proteins

**Figure 1:**
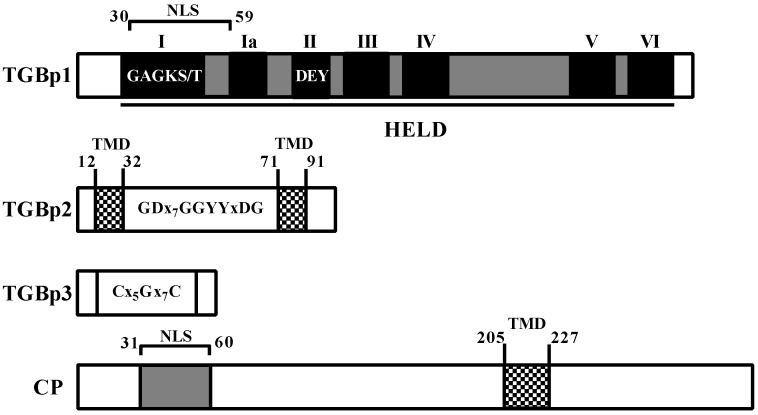
Sequence analysis of CYVCV proteins. The ‘HELD’ in TGBp1 referred to a helicase-like domain, and the numbers (I, Ia, II, III, IV, V, and VI) at the tops of dark boxes referred to seven motifs typical for a helicase. TMD, transmembrane domain as predicted using the ΔG prediction server. NLS, nucleus location signal.

The full-length cDNAs of genes encoding the TGBp1, TGBp2, TGBp3, CP, and 23K proteins of CYVCV-HB were cloned and verified by sequence analysis. These proteins of CYVCV-HB shared 96.6–100% identities with the corresponding proteins encoded by other CYVCV isolates available in GenBank (Table S5). Amino acid (aa) sequence alignments showed that TGBp1 contained a nucleus location signal (NLS) and a viral helicase 1 domain (HELD) covering almost the entire sequence. Within the domain, seven motifs (I, Ia, II, III, IV, V, and VI) were identified (Figure 1). The NTP-binding helicase motifs GAGKT (aa positions 30 to 34) and DEY (aa positions 76 to 78) for ATPase catalytic activity were highly conserved in all TGBp1 proteins of viruses in families *Alphaflexiviridae* and *Betaflexiviri*dae (i.e., fovea-, carla-, lola-, mandari-, and potexviruses). Five other motifs (Ia, III, IV, V and VI) also presented in the potex-like and hordei-like viruses [4]. TGBp2 contained two transmembrane domains (TMD) and a conserved central region GDx7GGxYxDG (Figure 1). TGBp3 contained three amino acid residues (Cys_27_, Gly_33_ and Cys_41_) that composed a Cx5Gx7C region (Figure 1), which was highly conserved in the TGBp3 proteins of viruses belonging to go the genera *Potexvirus*, *Carlavirus*, *Foveavirus* and *Allexivirus* [4]. The CYVCV CP harbored one NLS at aa positions 31–60 and one transmembrane domain at aa positions 205–223 (LALVVRDFCPLRAFCAYYSRVVW) as predicted using the ΔG prediction server (Figure 1). There was not any conserved domain identified in 23K protein.

### 3.2. Interactions of CYVCV Proteins Identified by Yeast Two-Hybrid (Y2H) Assay

**Figure 2:**
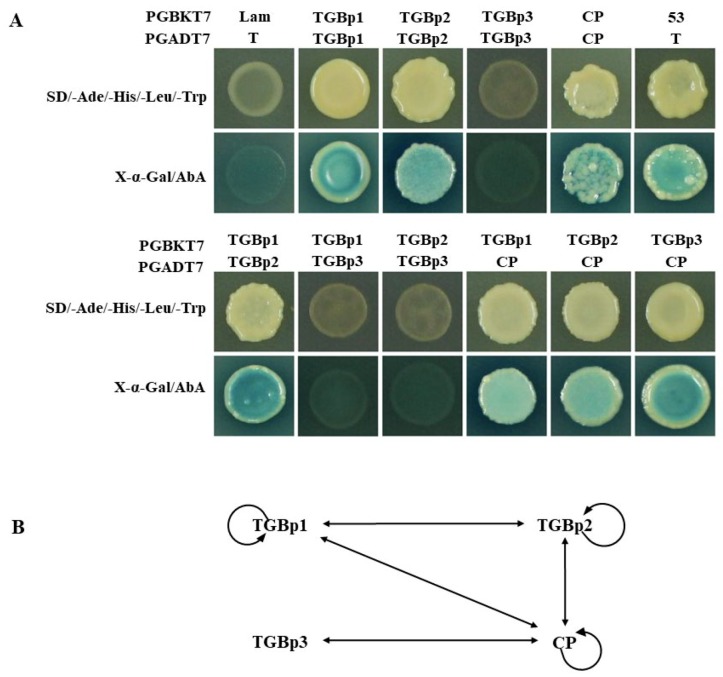
Yeast two-hybrid analysis of the protein-protein interactions of CYVCV. (**A**) Cells of a *Saccharomyces cerevisiae* strain Y2HGold were cotransformed with paired homologous proteins of TGBp1-TGBp1, TGBp2-TGBp2, TGBp3-TGBp3, CP-CP and heterologous proteins TGBp1-TGBp2, TGBp1-TGBp3, TGBp2-TGBp3, TGBp1-CP, TGBp2-CP, and TGBp3-CP. Their cDNAs were individually fused to pGBKT7 as bait plasmids (BD) and to pGADT7 as prey plasmids (AD). The pGBKT7-p53-pGADT7-T construct was used as a positive control, and pGBKT7-Lam-pGADT7-T was used as a negative control. Positive interactions were determined by the appearance of both white colonies and blue colonies on quadruple dropout agar medium SD/-Ade/-His/-Leu/-Trp (QDO) (upper panel) and QDO supplemented with X-α-Gal/AbA (lower panel), respectively. (**B**) A schematic showing the interaction relationship among CYVCV proteins as determined by Y2H. The circular arrowheads indicate the homologous interactions, and lines with double arrowheads indicate the heterologous interactions.

To assess potential heterologous and homologous interactions in vitro between CYVCV proteins, a yeast two-hybrid System (Clontech, Mountain View, CA, USA) was applied according to the manufacturer’s protocol. Results showed that the tested proteins in this system did not express any transcription activation domain in the absence of an interacting partner. Thereby, the potential interactions within each pair of the four proteins (TGBp1, TGBp2, TGBp3, and CP) were identified by using the Y2H system. The paired yeast cells containing bait and prey plasmids of TGBp1 and TGBp2 and CP genes survived on QDO culture medium (Figure 2A) and produced blue colonies on QDO culture media supplemented with 5-bromo-4-chloro-3-indolyl α-D-galactoside (X-α-Gal) (Figure 2A), suggesting self-interactions occurred within TGBp1, TGBp2, and CP but not within TGBp3. Potential interactions were observed between CP and each of the three TGB proteins and between TGBp1 and TGBp2 (Figure 2A,B). TGBp1-TGBp3 and TGBp2-TGBp3 did not exhibit a direct mutual interaction.

### 3.3. Intracellular Interactions of CYVCV Proteins

**Figure 3:**
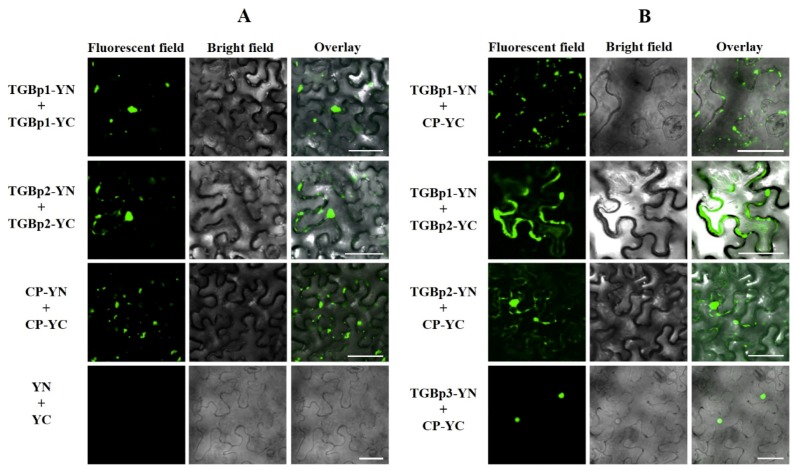
Bimolecular fluorescence (BiFC) assay of the protein-protein interactions of CYVCV in *N. benthamiana* epidermal cells. (**A**) Homologous interactions of CYVCV proteins TGBp1, TGBp2, and CP. (**B**) Heterogonous interactions of paired CYVCV proteins. The proteins were transiently coexpressed with recombinant vectors of BiFC (nYFP and cYFP) in *N. benthamiana* epidermal cells and visualized under confocal microscopy. YN and YC represent the C-terminus and N-terminus of YFP, respectively. The negative control with complementary empty vector tags is shown as “YN+YC”. Bar = 20 μm.

Bimolecular fluorescence complementation (BiFC) assays were conducted in *N. benthamiana* leaves. At 48 hpi, YFP fluorescence signals in infiltrated leaf cells were visualized under confocal microscopy. In accordance with results obtained in the Y2H assays, fluorescence signals were observed in the leaf cells coinfiltrated with the paired homologous proteins TGBp1-YN/TGBp1-YC, TGBp2-YN/TGBp2-YC and CP-YN/CP-YC (Figure 3A) and the heterogonous protein combinations TGBp1-YN/TGBp2-YC, CP-YC/TGBp1-YN, CP-YC/TGBp2-YN, and CP-YC/TGBp3-YN (Figure 3B). There was no fluorescent signal detected in leaf cells coinfiltrated with the paired homologous proteins YN/YC (negative control) (Figure 3A) and combinations TGBp3-YN/TGBp3-YC, TGBp1-YN/TGBp3-YC, and TGBp2-YN/TGBp3-YC (Figure S1). These results further confirmed the protein-protein interactions identified in the Y2H assays.

It was noticed that the subcellular localization of fluorescence signals produced by these combinations differed substantially. The CP and TGBp2 self-interaction emerged as numerous spots along the cell wall or granular structures in the cytoplasm, and the TGBp1 self-interaction mainly appeared as granular structures at the cell wall and in the cytoplasm (Figure 3A). The interaction between CP-YC and TGBp1-YN produced discontinuous punctate spots along the cell membrane (Figure 3B). CP-YC/TGBp2-YN interaction produced continuous or intermittent fluorescent signals in the cell periphery (Figure 3B). Moreover, the fluorescent signal of CP-YN/TGB3-YC interaction presented exclusively in the nucleus (Figure 3B).

### 3.4. Subcellular Localization of CYVCV Proteins in *N. benthamiana* Leaf Cells

**Figure 4:**
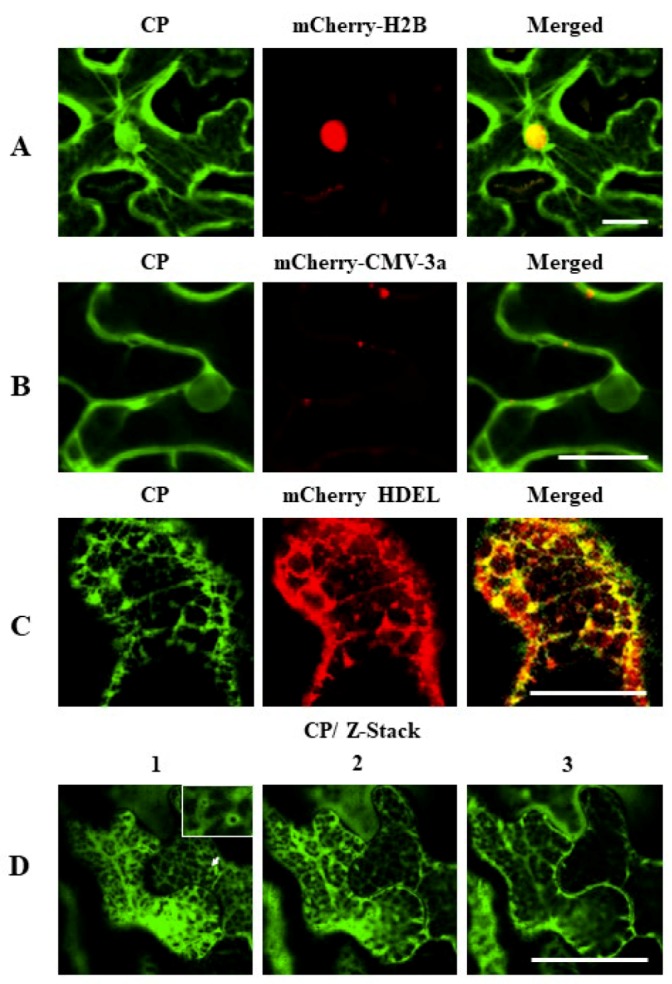
Subcellular location analyses of CYVCV coat protein (CP) in *N. benthamiana* epidermal cells. (**A**) Colocalization of CYVCV CP with marker protein mCherry-H2B at the nucleus. (**B**) CYVCV CP located at the nucleus and cell membrane but not colocalized with the PD marker protein mCherry-CMV-3a. (**C**) Colocalization of CYVCV CP with marker protein mCherry-HDEL at the ER. (**D**) Z-stack images of CYVCV CP in *N. benthamiana* epidermal cells captured at different layers (1–3). The vesicle-like structures developed from the ER are highlighted in the box (D1). Scale bars = 50 μm.

The subcellular localization of CYVCV proteins in the epidermal cells of *N. benthamiana* leaves were determined by using the transient expression vector pCNY, and fluorescence signals in infiltrated leaf cells were visualized under confocal microscopy. When YFP was expressed alone, the fluorescence signals of free YFP were observed in the cytoplasm and nucleus almost uniformly, as well as in actin filaments close to nucleus (Figure S2). The fluorescence signal of pCNY-CP was observed in the nucleus, cell membrane and fiber forms (Figure 4A). The nuclear localization of CP was validated by using mCherry-H2B, a nuclear marker protein. However, CP was not colocalized with the PD marker protein mCherry-CMV-3a (Figure 4B). When green fluorescence signals were captured at lower leaf cell layers, the fluorescence signal was also observed to accumulate in the endoplasmic reticulum (ER), and cytoplasm and notably overlapped with the red fluorescence released by the ER marker mCherry-HDEL (Figure 4C). Moreover, vesicle-like structures appeared to bud from the ER membrane (Figure 4D), indicating that CP might be associated with the ER and ER-derived vesicles.

**Figure 5:**
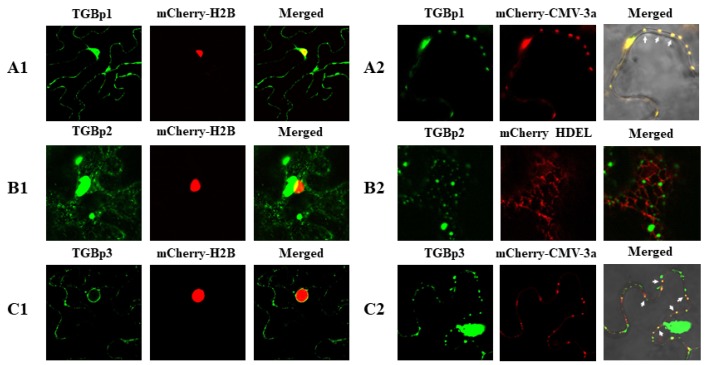
Subcellular localization analysis of CYVCV triple gene proteins (TGBp1, TGBp2 and TGBp3) in *N. benthamiana* epidermal cells. (**A1**) and (**A2**) Colocalization of CYVCV-TGBp1 with mCherry-H2B and mCherry-CMV3a at the nucleus and PD. (**B1**) and (**B2**) Colocalization analysis of CYVCV-TGBp2 with nuclear marker mCherry-H2B and ER marker mCherry-HDEL. (**C1**) and (**C2**) Colocalization of CYVCV-TGBp3 with mCherry-H2B at the nuclear membrane (perinucleus) and plasmodesmata (PD). Arrows indicated discontinuous punctate spots where CYVCV TGBp1 and TGBp3 colocalized with PD marker mCherry-CMV-3a. Photos were taken at 48 h post infiltration (hpi).

The fluorescence signals released by the three diffused pCNY-TGBps clearly differed in their subcellular locations. Of those, the fluorescent signal of diffused pCNY-TGBp1 was observed as punctate spots along the cell membrane and accumulated in the nuclear periplasm but was absent in the nucleolus. Further tests confirmed that CYVCV-TGBp1 colocalized with the nuclear marker protein mCherry-H2B and the PD marker protein mCherry-CMV-3a at the nucleus (Figure 5(A1)) and PD (Figure 5(A2)), respectively. The nuclear location of TGBp1 was in agreement with a predicted moderately strong bipartite NLS (cNLS mapper score 3.8) in the protein (Figure 1). The green fluorescence of pCNY-TGBp2 accumulated as granular bodies in ER or along the ER nets and as a sizable amorphous mass often seen near the nucleus (Figure 5(B1)). However, when pCNY-TGBp2 coexpressed with the nuclear marker protein mCherry-H2B (Figure 5(B1)) and the ER marker protein mCherry-HDEL (Figure 5(B2)), it was found that granular-like structures were embedded within ER networks, but not labeled with the nuclear marker. The pCNY-TGBp3 fluorescence signals appeared as discontinuous punctate spots in the cell periphery and also accumulated at the perinuclear region (Figure 5(C1)). However, the green florescence surrounded the nucleus but was not colocalized with mCherry-H2B at the nucleus (Figure 5(C1)). The discontinuous punctate spots in the cell periphery were well labeled with the PD marker protein (Figure 5(C2)).

**Figure 6:**
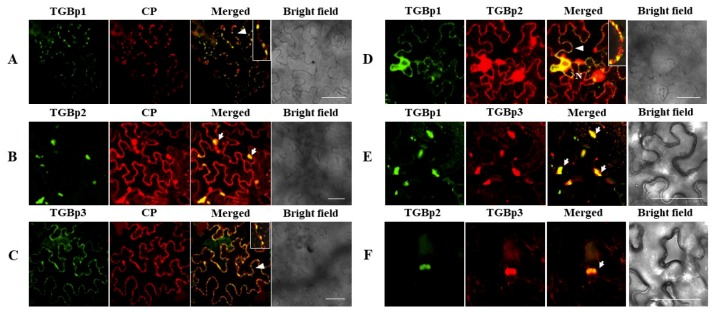
Subcellular colocalization analyses of CYVCV proteins fused with CFPs (green fluorescence) and YFPs (red fluorescence) by transient expression in *N. benthamiana* leaves and visualization under confocal microscopy. (**A**–**C**) Colocalization of CYVCV CP with TGBp1, TGBp2, and TGBp3 at the PD, granular masses and PD, respectively. (**D**–**F**) Colocalization of CYVCV paired proteins TGBp1/TGBp2, TGBp1/TGBp3 and TGBp2/TGBp3. White arrows indicate colocalization as aggregates. The PD colocalization (white arrowheads), is highlighted in the boxes at the right upper corner of the corresponding figure panels. Photos were taken at 72 hpi. Scale bar = 25 μm.

The colocalization features of CYVCV proteins were examined by individually coexpressing each pair of proteins in epidermal cells of *N. benthamiana* leaves. Cells cotransformed with expression plasmids of paired proteins (TGBp1/CP, TGBp2/CP, TGBp3/CP, TGBp1/TGBp2, TGBp1/TGBp3, and TGBp2/TGBp3), which were labeled with CFP and YFP, were examined under confocal microscopy. The signal associations were observed for all these combinations (Figure 6). The results were concordant with the interaction relationships of pairwise proteins as tested by BiFC assays. Importantly, punctate red and green fluorescent signals expressed in combinations of pCNC-TGBp1/pCNY-CP, pCNC-TGBp3/pCNY-CP, and pCNC-TGBp1/pCNY-TGBp2 were observed at opposite positions along the membranes of adjacent cells (Figure 6A,C,D). The results indicated that the paired proteins might colabel the PD of epidermal cells of *N. benthamiana* leaves. Notably, pCNC-TGBp2 alone was localized as granular bodies within ER nets (Figure 5(B1,B2)), but the expressed fluorescence signal of pCNC-TGBp2 was translocated to the PD when it was coexpressed with pCNY-TGBp1 in leaf cells (Figure 6D). Similarly, the expressed fluorescence signal of pCNC-CP was translocated to the PD when it was coexpressed with pCNC-TGBp1 or pCNC-TGBp3 (Figure 6A,C). These findings are consistent with the BiFC results (Figure 3B), further confirming the *in planta* interactions. The fluorescence of pCNY-CP and pCNC-TGBp2 coaccumulated in granular-like structures in the cytoplasm (Figure 6B) but not in the nucleus, suggesting that the presence of TGBp2 could affect the subcellular localization of CP by recruiting CP to the peripheral bodies. The coexpression of pCNC-TGBp3 and pCNY-CP showed a substantial proportion of fluorescence appearing as foci localized at the PD along the cell wall (Figure 6C), differing from their nuclear localization during their in planta interaction, as revealed by BiFC assays (Figure 3B). Additionally, the paired proteins TGBp1-TGBp3 and TGBp2-TGBp3 were included in the assay. The results showed that when pCNY-TGBp3 was coinfiltrated with pCNC-TGBp1 or pCNC-TGBp2, their fluorescence signals partially colocalized as granular bodies (Figure 6E,F).

### 3.5. CYVCV CP Functions as an RSS

**Figure 7:**
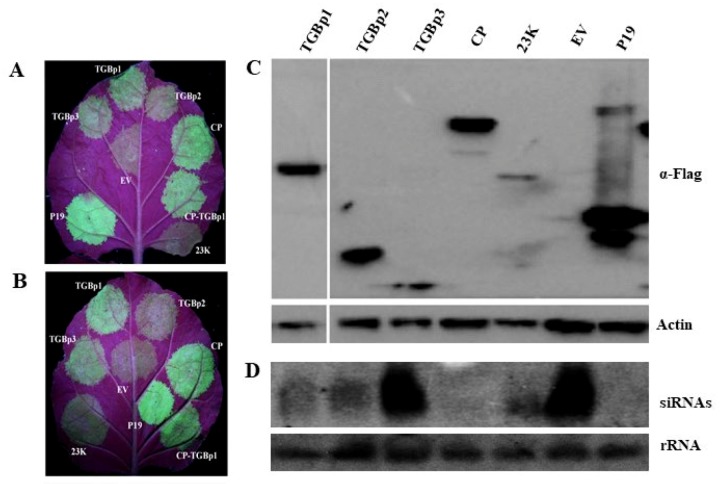
Identification of local RNA silencing suppression activity of CYVCV proteins. (**A**,**B**) Leaves of wild-type *N. benthamiana* plants were coinfiltrated with recombinant plasmids and visualized under long-wavelength UV light at 4 dpi. Leaf patches coinfiltrated with tomato bushy stunt virus (TBSV) P19 and 35S-GFP or an empty vector (EV) and 35S-GFP were used as positive or negative controls, respectively. (**C**,**D**) Western and Northern blot analyses of GFP and gfp-siRNA in infiltrated blotches of *N. benthamiana* leaves, respectively. Actin and rRNA from the same *N. benthamiana* samples were used as loading controls.

To identify potential local RNA RSSs encoded by CYVCV, the candidate CYVCV genes encoding TGBp1, TGBp2, TGBp3, CP, and 23K were individually inserted into the binary pCNF3 vector and coexpressed with PMS4 (35S-gfp) in leaf cells of wild-type *N. benthamiana* plants by using an agroinfiltration assay [47]. *Agrobacterium* cultures harboring an empty vector (EV) or a vector with the RSS P19 of TBSV were used as a negative or positive control, respectively. The green fluorescence signals were observed under long-wavelength UV light. When the empty vector was co-agroinfiltrated with 35S-gfp, GFP fluorescence signal in the infiltrated leaf patches peaked at two or three days post infiltration (dpi) and decreased thereafter and was undetectable or very weak at 4 or 5 dpi due to the induction of RNA silencing. In contrast, the coinfiltration of *Agrobacterium* cultures containing CYVCV CP and 35S-gfp produced intensive GFP (green fluorescence), which was comparable to that of P19 infiltration (Figure 7A). The fluorescence signal could last at 6 dpi and disappeared thereafter. The results indicated that CYVCV CP could efficiently suppress posttranscriptional RNA silencing. Leaf patches individually coinfiltrated with TGBp1/35S-gfp and TGBp3/35S-gfp produced relatively weak green fluorescence signals at 4 dpi. TGBp2/35S-gfp infiltration occasionally produced a very weak green fluorescence signal, but 23K could not enhance the signal (Figure 7A). Furthermore, when equal volumes of TGBp1 and CP were coinfiltrated with 35S-gfp, the intensity of green fluorescence signal was identical to that produced by single CP/35S-gfp infiltration, indicating that TGBp1 did not affect the RNA silencing suppression activity of CP (Figure 7A,B). Additional tests to investigate the potential of CYVCV proteins for inhibiting systemic RNA silencing revealed that all five proteins failed to block systemic silencing signals (Figure S3).

Furthermore, the transient expression levels of both CYVCV proteins and small interference RNAs of GFP (gfp-siRNA) in infiltrated leaf patches of *N. benthamiana* plants at 4 dpi were tested by western blot and northern blot analyses, respectively. The Western blot assays showed that all five proteins were successfully expressed; however, TGBp3 and 23K had relatively lower expression levels (Figure 7C). The Northern blot assays showed that the hybridization signals for gfp-siRNA in extracts of CP/35S-gfp- and P19/35S-gfp-infiltrated tissues were very weak or undetectable (Figure 7D). The result correlated with the extensive GFP fluorescence signals as shown in Figure 7A. These results suggested that CYVCV CP was a strong RSS. In TGBp3/35S-gfp-infiltrated leaf tissues, high levels of gfp-siRNA accumulation were detected, which were identical to those produced by treatment with the empty vector control. Compared to those of TGBp3/35S-gfp, gfp-siRNA hybridization signals produced in TGBp1/35S-gfp, TGBp2/35S-gfp, and 23K/35S-gfp infiltrations were relatively lower but were significantly higher than those in CP/35S-gfp- and P19/35S-gfp-infiltrated tissues (Figure 7D).

## 4. Discussion

Recent studies on the interactions of TGB proteins and CP have made it possible to generate a model for the cell-to-cell movement of potexviruses and hordeiviruses in plants [48,49]. However, the protein-protein interaction has never been reported for viruses in the genus *Mandarivirus.* Here, we showed that CYVCV CP, TGBp1 and TGBp2 are all self-interacting, which is similar to those of PVX and other potexviruses [10,12,26,28,50]. Previous BiFC assays showed that TGBp2 of BaMV could interact with TGBp1 and TGBp3, and its CP could interact with TGBp1 and TGBp2 [10,26]. For PVX, its TGBp2 and TGBp3 interacted with each other, while TGBp3 was not associated with CP in the BiFC assays [26]. Notably, the CYVCV TGBp3 showed a specific interaction relationship with CP but not with TGBp2 in both Y2H and BiFC assays, distinctly different from the results of previous studies on potexviruses [12,51]. The reason for the difference between CYVCV and PVX regarding their TGBp2 and TGBp3 interactions might be due to the divergent sequence features between CYVCV-TGBp3 and PVX-TGBp3. PVX-TGBp3 possessed a transmembrane domain at its N-terminus (aa sites 6 to 26), but it was absent in the CYVCV-TGBp3 (Figure S4A). Their folding structures were also different (Figure S4B). Yet, further studies will shed light on the found differences.

Determination of the subcellular localization of viral proteins is an important step in revealing their putative functions during virus infection. The subcellular localization of viral proteins could provide clues to understand the interaction between viral proteins and host factors and their functions during virus infection. The TGBp1 of some potex-like viruses function in the disassembly of virions and vRNA translation [19,20,49]. Similar to its counterparts in some potex-like viruses [11,15,52,53,54] and hordei-like PMTV [6,55,56], CYVCV TGBp1 contained all seven motifs conserved in ATPase/helicases and a nuclear location signal (NLS). As expected with the presence of an NLS, TGBp1 localized in the nucleoplasm. Additionally, the protein well-localized at the PD. Many plant RNA viruses and their movement proteins are associated with the ER network of their hosts, and the plant ER is involved in plant virus movement and viral replication complex formation [3,41,57,58,59,60]. The TGB proteins of some plant viruses were also found to target the plasmodesmata by association with the ER [4,26,48]. As many integral membrane-associated proteins, CYVCV TGBp2 has two transmembrane helices. However, the protein was localized within the ER networks but not at the ER. This observation was different from that of potexvirus TGBp2, which localized to the ER [22,27]. Although the transmembrane domain necessary for membrane anchoring [57] was absent in CYVCV TGBp3 compared with the TGBp3 of potexviruses [61], CYVCV TGBp3 exhibited locations at the nuclear membrane and cell PD. Viral CPs perform multiple functions in virus infection [24]. CYVCV CP alone localized to the nucleus and ER network in *N. benthamiana* epidermal cells. However, the biological function of the ER location of CYVCV CP remains to be explored in further studies. The interaction dynamics of viral movement proteins might affect their localization, as was found for potato leafroll virus (PLRV) [62]. The TGBp3 of potexvirus targeted TGBp2 and formed peripheral puncta in close proximity to the plasmodesmata [13,61]. The PD localization of BaMV TGBp1 required assistance from both TGBp2 and TGBp3 and the TGBp1, TGBp2, and TGBp3 coordinately function on facilitating the viral movement in host plants [3]. However, different from BaMV TGBp1, we found here that CYVCV TGBp1 alone exhibited PD localization and could direct the PD localization of viral CP and TGBp2; viral TGBp3 also directed CP to the PD. Thus, the interactions of TGBp1 and TGBp3 with CP likely play roles in locating CP to the PD. The results indicated that the TGB proteins-CP complex and PD locations of these proteins might be necessary for the viral intracellular transport, as observed for potexviruses [26,52,63,64]. CYVCV is a newly characterized virus. Like most viruses infecting woody plants, it is usually very difficult to study viral protein functions due to the limitation of transmission. We tried to construct infectious clones of CYVCV, but they failed to infect *N. benthamiana* and other herbaceous plant species. Recently, another research group in China obtained CYVCV infectious clones that can infect *Citrus sinensis* (L) Osbeck seedlings through an *Agrobacterium*-mediated vacuum-infiltration strategy but that failed to infect *N. benthamiana* plants [65]. Additionally, CYVCV could be mechanically transmitted to several herbaceous plants with low efficiency. Thus, there is currently a technical obstacle to study the protein functions of CYVCV. Our study of the protein-protein interactions and protein subcellular localizations of CYVCV provided new insights into further exploring the functions of these proteins in the viral movement.

RNA silencing is an important plant immune mechanism against virus infection [66]. To overcome RNA silencing of the host, many plant viruses encode one or more RSS proteins [67]. Currently, TGBp1 has been identified to be an efficient RSS for several potexviruses [33,34,68]. Among all TGB-encoding plant viruses, both CP and TGBp1 of PepMV have been reported to serve as RSS proteins [24]. In CYVCV-infected plants, the accumulation of viral siRNA has been reported [40], indicating the occurrence of RNA silencing in the host to defend against the virus. The data presented here showed, for the first time, that the CP of CYVCV functioned to counteract RNA silencing and was able to mediate local (intracellular) suppression of RNA silencing. In consistent with the result, a parallel study of our group showed that apple stem pitting virus (ASPV, genus *Foveavirus*) can suppress local silencing in *N. benthamiana*, but cannot suppress systemic silencing [69]. To our knowledge, this was the third case of CP acting as an RSS in TGB-encoding plant viruses. Similar to the TGBp1 of other potexviruses, CYVCV TGBp1 possesses an NLS signal and RNA-binding activity and could interact with CP. However, in tissues coexpressing CYVCV TGBp1 with 35S-gfp, fluorescence signals were maintained at 4 dpi but were significantly weaker than those induced by CP, which were concordant with their gfp-siRNA levels in the infiltrated tissues. Then, similar to PepMV [24], CYVCV CP was a more efficient RSS than TGBp1. The RNA silencing suppressor activity of TGBp1 exhibits remarkable variability among potexviruses [34,70]. Further studies will be necessary for exploring the silencing suppression mechanism of CYVCV CP.

In conclusion, this study presents the first report of interaction and subcellular localization relationships of the CP and three TGB proteins of a mandarivirus. In difference with potexviruses, CYVCV TGBp3 interacted with CP but not with TGBp2; its TGBp1 located at PD without the help of viral TGBp3. We found here that CYVCV TGBp1 could direct the PD localization of viral CP and TGBp2; viral TGBp3 also directed CP to the PD. Our study revealed that CYVCV CP had RSS activity for the first time. The findings extend the knowledge of a protein interaction model and CP functions in a mandarivirus.

## Supplementary Materials

The following are available online at https://www.mdpi.com/1999-4915/11/4/329/s1, Figure S1: Bimolecular fluorescence assay (BiFC) assay of combinations TGBp3-YN/TGBp3-YC, TGBp1-YN/TGBp3-YC and TGBp2-YN/TGBp3-YC of CYVCV in *N. benthamiana* epidermal cells. Figure S2: YFP was expressed alone showing free YFP in the cytoplasm, nucleus, and actin filaments close to the nucleus in *N. benthamiana* epidermal cells. Figure S3: Systemic RNA silencing suppression assays of five proteins encoded by CYVCV. Leaves of *N. benthamiana* 16c plants were coinfiltrated with recombinant plasmids. An empty vector (SPDK658-dTGBdCP) was used as a negative control. Tomato bushy stunt virus (TBSV) p19 was used as a positive control. Images were taken under long-wavelength UV light at 20 days post inoculation (dpi). Figure S4: The predicted graphic depiction of the transmembrane domains of CYVCV-TGBp3 and PVX-TGBp3 (A) and three-dimensional structures of CYVCV-TGBp3 (green) and PVX-TGBp3 (brown), and their superimposition (B). Table S1: The primers used in PCR amplification of CYVCV genomic ORF 2-6. Table S2: Primers used to construct entry vectors for BiFC experiments. Table S3: Primers used to construct vectors for subcellular localization experiments and for the identification of local RNA silencing suppressors. Table S4: Primers used to construct vectors for the identification of systemic RNA silencing suppressors. Table S5: Nucleotide and amino acid sequence similarities of the cDNAs of open reading frame (ORF) 2–6 between CYVCV-HB and isolates available in GenBank.
