# Supplementary material for: The Coat Protein of Citrus Yellow Vein Clearing Virus Interacts with Viral Movement Proteins and Serves as an RNA Silencing Suppressor
Source: Viruses. 2019 Apr 5;11(4):329. doi: 10.3390/v11040329 (PMC6520955; doi:10.3390/v11040329)
Supplement: Supplementary file 1 [file viruses-11-00329-s001.zip › supplementary materials/Supplementary Figure.docx]

**
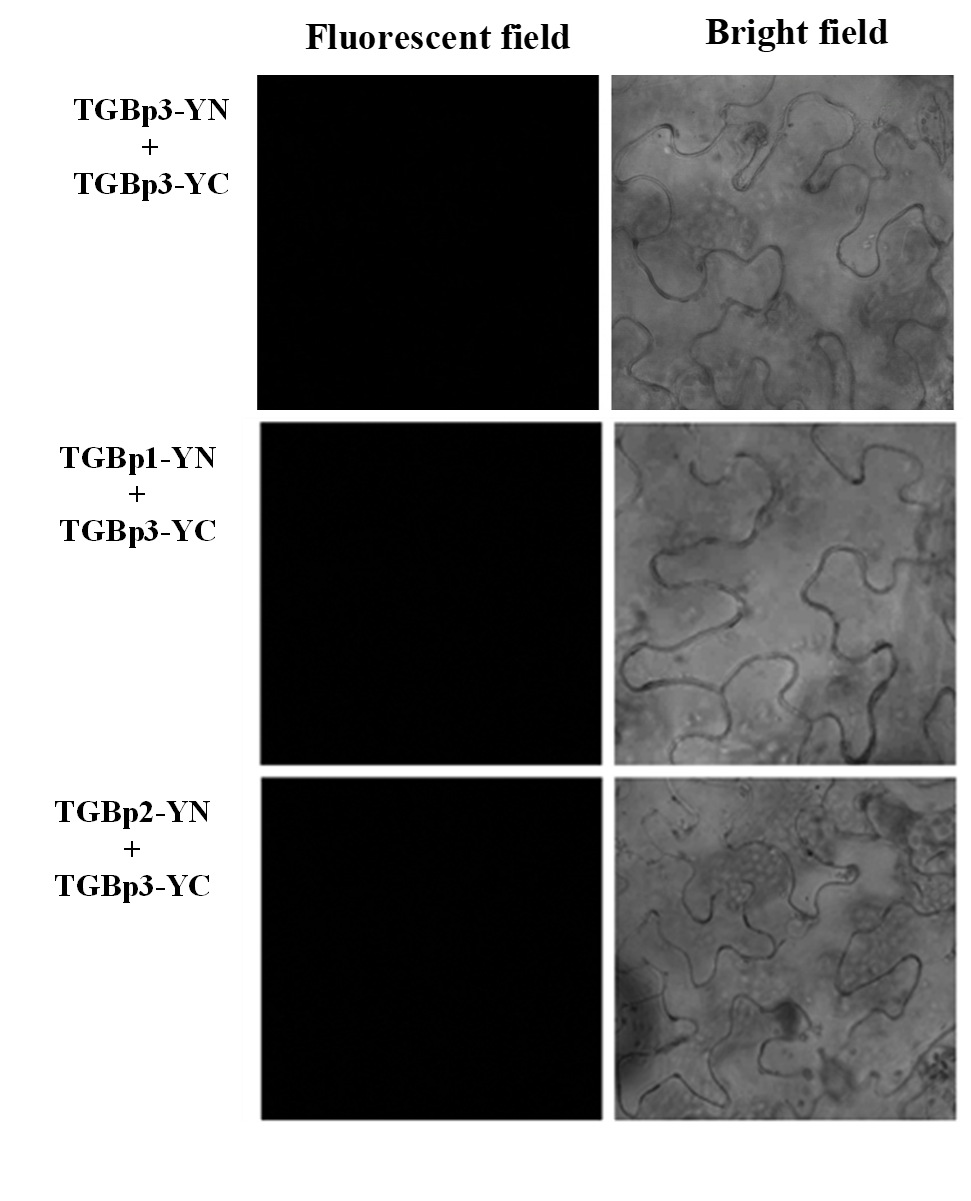
**

**Figure S1**. Bimolecular fluorescence assay (BiFC) assay of combinations TGBp3-YN/TGBp3-YC, TGBp1-YN/TGBp3-YC and TGBp2-YN/TGBp3-YC of CYVCV in *N. benthamiana* epidermal cells.


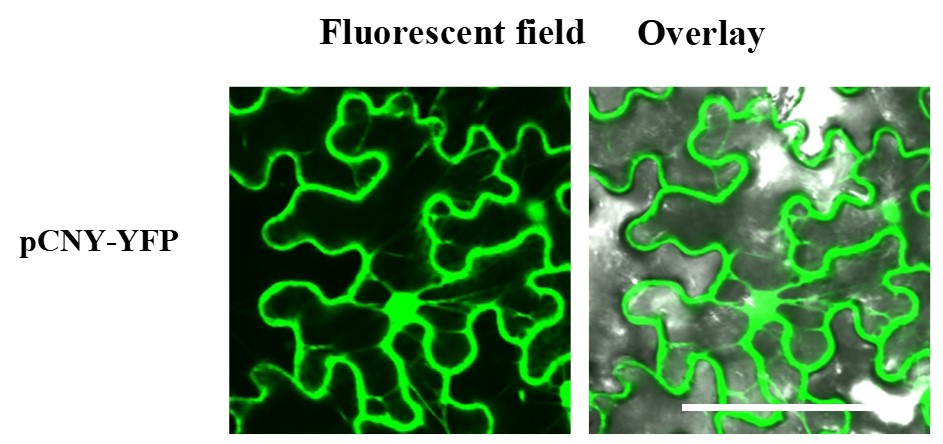


**Figure S2.** YFP was expressed alone showing free YFP in the cytoplasm, nucleus and actin filaments close to the nucleus *N. benthamiana* epidermal cells.


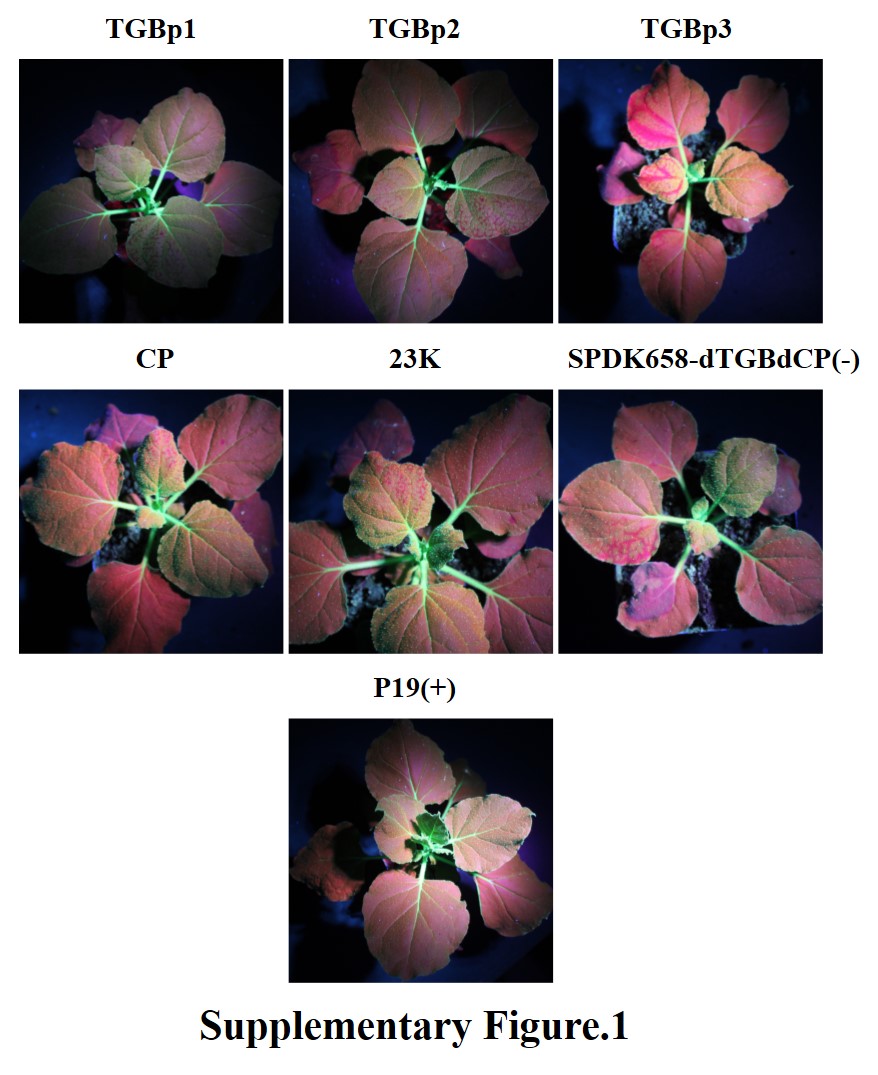


**Figure S3**. Systemic RNA silencing suppression assays of five proteins encoded by CYVCV. Leaves of *N. benthamiana* line 16c plants were co-infiltrated with recombinant plasmids. An empty vector (SPDK658-dTGBdCP) was used as a negative control. Tomato bushy stunt virus (TBSV) p19 was used as a positive control. Images were taken under long wavelength UV light at 20 days post inoculation (dpi).

**A**

**CYVCV-TGBp3**

**PVX-TGBp3**

**CYVCV-TGBp3**

**PVX-TGBp3**

**CYVCV-TGBp3**

**+**

**PVX-TGBp3**

**B**


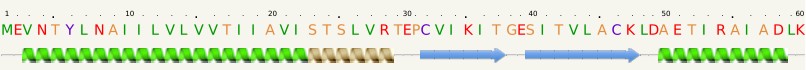

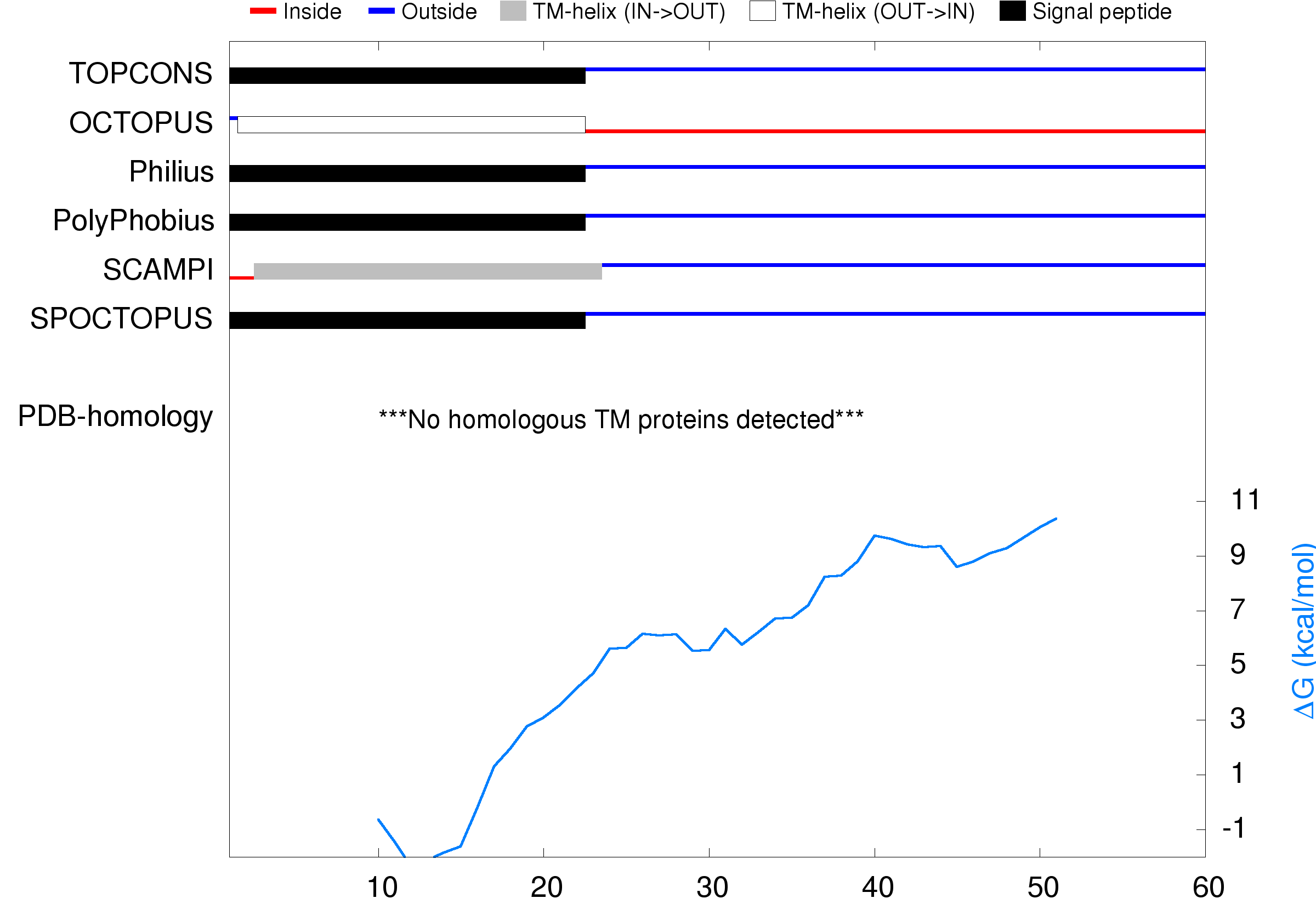

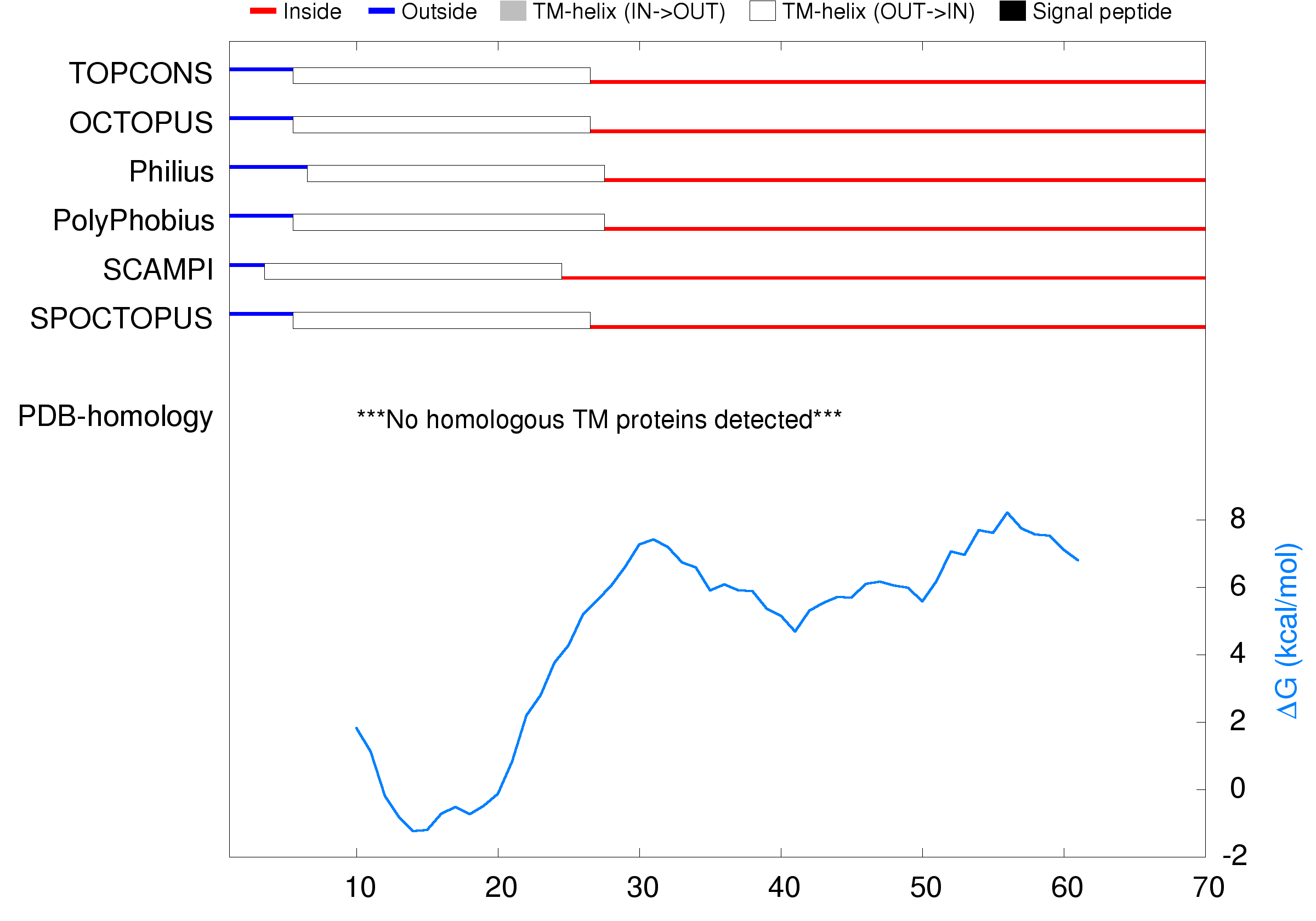

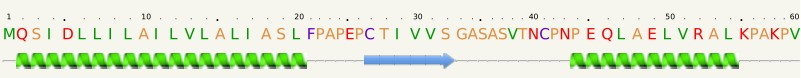


**TM-helix**

Phyre2

algorithm

Phyre2

algorithm

**Figure S4.** The predicted graphic depiction of the transmembrane domains of CYVCV-TGBp3 and PVX-TGBp3 (A) and three-dimensional structures of CYVCV-TGBp3 (green) and PVX-TGBp3 (brown), and their superimposition (B).
