# Supplementary material for: The Coat Protein of Citrus Yellow Vein Clearing Virus Interacts with Viral Movement Proteins and Serves as an RNA Silencing Suppressor
Source: Viruses. 2019 Apr 5;11(4):329. doi: 10.3390/v11040329 (PMC6520955; doi:10.3390/v11040329)
Supplement: Supplementary file 1 [file viruses-11-00329-s001.zip › supplementary materials/Supplementary Table.docx]

Table S1. **The primers used in PCR amplification of CYVCV genomic ORFs 2-6**

| **ORFs** | **Primers (5´-3´)** | **Enzyme** | **Size (bp/aa)** |
| --- | --- | --- | --- |
| TGB1 | F: CGGAATTCATGGACTTACCTGAGCTCCTG | *EcoR* I | 678/226 |
|  | R: CGGGATCC TCAGGAGGAGGTTGTAGAGGCA | *Bam*H I |  |
| TGB2 | F: CGGAATTCATGCCTCTACAACCTCCTCCT | *EcoR* I | 327/109 |
|  | R: CGGGATCCTCAGGTGCGGGGAATAGGCTG | *Bam*H I |  |
| TGB3 | F: CGGAATTCATGCAGTCAATAGATTTACTAATCCTC | *EcoR* I | 183/61 |
|  | R: CGGGATCCTTAAACCGGTTTGGCCGGTTT | *Bam*H I |  |
| CP | F: CGGAATTCATGAGCTTCGACTACACTCAC | *EcoR* I | 978/325 |
|  | R: CGGGATCCTTAGATGTTGAAAGGGGTCGGG | *Bam*H I |  |
| 23K | F: CGGAATTCATGGAACCTCATGATCAAGGC | *EcoR* I | 669/223 |
|  | R: CGGGATCCTCATCTGGGGTCAAGGAGCTC | *Bam*H I |  |

The enzyme digestion sites were underlined.

Table S2. **Primers used to construct entry vectors for BiFC experiments**

| **Target gene** | **Primer name** | **Sequence(5´-3´)** | **Product size (**bp) |
| --- | --- | --- | --- |
| TGB1 | attb-TGB1-F | AAAAAGCAGGCTCCATGGACTTACCTGAGCTCCTG | 678 |
|  | attb-TGB1-R | AGAAAGCTGG GTAGGAGGAGGTTGTAGAGGCA |  |
| TGB2 | attb-TGB2-F | AAAAAGCAGGCTCCATGCCTCTACAACCTCCTCCT | 327 |
|  | attb-TGB2-R | AGAAAGCTGGGTAGGTGCGGGGAATAGGCTG |  |
| TGB3 | attb-TGB3-F | AAAAAGCAGGCTCCATGCAGTCAATAGATTTACTAATCCTC | 183 |
|  | attb-TGB3-R | AGAAAGCTGGGTAAACCGGTTTGGCCGGTTT |  |
| CP | attb-CP-F | AAAAAGCAGGCTCCATGAGCTTCGACTACACTCAC | 978 |
|  | attb-CP-R | AGAAAGCTGGGTAGATGTTGAAAGGGGTCGGG |  |
|  | attB-1 | GGGGACAAGTTTGTACAAAAAAGCAGGCT |  |
|  | attB-2 | GGGGACCACTTTGTACAAGAAAGCTGGGT |  |

The *attB* recombination sites were underlined.

Table S3. **Primers used to construct vectors for subcellular localization experiments and the identification of local RNA silencing suppressor**

| **ORFs** | **Primers (5´-3´)** | **Enzyme** | **Size (bp/aa)** |
| --- | --- | --- | --- |
| TGB1 | F: TGCTCTAGA ATGGACTTACCTGAGCTCCTG | *Xba* I | 678/226 |
|  | R: CGGGATCC TCAGGAGGAGGTTGTAGAGGCA | *Bam*H I |  |
| TGB2 | F: TGCTCTAGA ATGCCTCTACAACCTCCTCCT | *Xba* I | 327/109 |
|  | R: CGGGATCCTCAGGTGCGGGGAATAGGCTG | *Bam*H I |  |
| TGB3 | F: TGCTCTAGA ATGCAGTCAATAGATTTACTAATCCTC | *Xba* I | 183/61 |
|  | R: CGGGATCCTTAAACCGGTTTGGCCGGTTT | *Bam*H I |  |
| CP | F: TGCTCTAGA ATGAGCTTCGACTACACTCAC | *Xba* I | 978/325 |
|  | R: CGGGATCCTTAGATGTTGAAAGGGGTCGGG | *Bam*H I |  |
| 23K | F: TGCTCTAGA ATGGAACCTCATGATCAAGGC | *Xba* I | 669/223 |
|  | R: CGGGATCCTCATCTGGGGTCAAGGAGCTC | *Bam*H I |  |

Table S4. **Primers used to construct vectors for the identification of systemic RNA silencing suppressor.**

| **Target Gene** | **Primer Name** | **Sequence(5´-3´)** |
| --- | --- | --- |
| TGB1 | spdk-TGB1-F | GCACCAGCTAGCATCGATTGGCGCGATGGACTTACCTGAGCTCCTG |
|  | spdk-TGB1-R | AGAAAGCTGG GTAGGAGGAGGTTGTAGAGGCA |
| TGB2 | spdk-TGB2-F | GCACCAGCTAGCATCGATTGGCGCGATGCCTCTACAACCTCCTCCT |
|  | spdk-TGB2-R | AGAAAGCTGGGTAGGTGCGGGGAATAGGCTG |
| TGB3 | spdk-TGB3-F | GCACCAGCTAGCATCGATTGGCGCGATGCAGTCAATAGATTTACTAATCCTC |
|  | spdk-TGB3-R | AGAAAGCTGGGTAAACCGGTTTGGCCGGTTT |
| CP | spdk-CP-F | GCACCAGCTAGCATCGATTGGCGCGATGAGCTTCGACTACACTCAC |
|  | spdk-CP-R | AGAAAGCTGGGTAGATGTTGAAAGGGGTCGGG |
| 23K | Spdk-23K-F | GCACCAGCTAGCATCGATTGGCGCGATGGAACCTCATGATCAAGGC |
|  | Spdk-23K-R | CGGTCGACGCGGCCGCTCCAGGCGCG TCATCTGGGGTCAAGGAGCTC |

**Table S5. Nucleotide and amino acid sequence similarities of the cDNAs of open reading frame (ORF) 2 to 6 between CYVCV-HB and isolates available in GenBank.**

| **Isolate (accession no.)** | **ORF2** | | **ORF3** | | **ORF4** | | **ORF5** | | **ORF6** | |
| --- | --- | --- | --- | --- | --- | --- | --- | --- | --- | --- |
|  | nt% | aa% | nt% | aa% | nt% | aa% | nt% | aa% | nt% | aa% |
| CYVCV-Y1 (JX040635) | 96.5 | 97.8 | 99.7 | 98.1 | 97.8 | 96.7 | 96.8 | 96.9 | 98.1 | 97.3 |
| CYVCV-RL (KP120977) | 98.5 | 99.1 | 99.7 | 99.1 | 98.9 | 100 | 98.6 | 98.5 | 99.0 | 98.2 |
| CYVCV-PK (KP313241) | 97.1 | 98.2 | 99.4 | 100 | 98.9 | 100 | 97.0 | 96.6 | 98.2 | 97.7 |
